# External Validation of an Artificial Neural Network and Two Nomograms for Prostate Cancer Detection

**DOI:** 10.5402/2012/643181

**Published:** 2012-07-05

**Authors:** Thorsten H. Ecke, Steffen Hallmann, Stefan Koch, Jürgen Ruttloff, Henning Cammann, Holger Gerullis, Kurt Miller, Carsten Stephan

**Affiliations:** ^1^Department of Urology, HELIOS Hospital, 15526 Bad Saarow, Germany; ^2^Institute of Pathology, HELIOS Hospital, Bad Saarow, Germany; ^3^Institute of Medical Informatics, Charité—Universitätsmedizin Berlin, 10098 Berlin, Germany; ^4^Department of Urology, Lukas Hospital Neuss, Germany; ^5^Department of Urology, Charité—Universitätsmedizin Berlin, 10098 Berlin, Germany

## Abstract

*Background*. Multivariate models are used to increase prostate cancer (PCa) detection rate and to reduce unnecessary biopsies. An external validation of the artificial neural network (ANN) “ProstataClass” (ANN-Charité) was performed with daily routine data. *Materials and Methods*. The individual ANN predictions were generated with the use of the ANN application for PSA and free PSA assays, which rely on age, tPSA, %fPSA, prostate volume, and DRE (ANN-Charité). Diagnostic validity of tPSA, %fPSA, and the ANN was evaluated by ROC curve analysis and comparisons of observed versus predicted probabilities. *Results*. Overall, 101 (35.8%) PCa were detected. The areas under the ROC curve (AUCs) were 0.501 for tPSA, 0.669 for %fPSA, 0.694 for ANN-Charité, 0.713 for nomogram I, and 0.742 for nomogram II, showing a significant advantage for nomogram II (*P* = 0.009) compared with %fPSA while the other model did not differ from %fPSA (*P* = 0.15 and *P* = 0.41). All models overestimated the predicted PCa probability. *Conclusions*. Beside ROC analysis, calibration is an important tool to determine the true value of using a model in clinical practice. The worth of multivariate models is limited when external validations were performed without knowledge of the circumstances of the model's development.

## 1. Introduction

Prostate specific antigen (PSA) is the most valuable tool for prostate cancer (PCa) detection [1]. The status of digital rectal examination (DRE) remains important, but especially for PCa screening the DRE is less important than PSA [2]. Transrectal-ultrasound- (TRUS-) guided needle biopsy of the prostate is nowadays the most simple and accurate method to obtain prostatic tissue for histological evaluation [3]. Although PSA is regarded as the best biochemical marker for PCa [4], an important limitation regarding its use in cancer detection is the considerable overlap of patients with PCa and those with benign prostate hyperplasia (BPH), specifically in the serum PSA range 4.0–10.0 ng/mL [4]. Percent-free PSA (%fPSA) has been proposed as a primary decision tool for first-time biopsy in men with a nonsuspicious DRE within the tPSA range 4–10 ng/mL, as well as in lower PSA values [5, 6]. 

Beside nomograms [7–9], artificial neural networks (ANNs) represent a main tool to help the clinician in risk stratifying the probability of PCa at needle biopsy [10–12]. 

Many studies using receiver operator characteristic (ROC) curve analysis on different classification models like logistic-regression- (LR-) based nomograms or ANN have been recently reviewed [13–15]. Chun et al. [16] compared the LR-based nomogram [8] and ANN [10] and showed similar performance of both models (AUC 0.71 and 0.737) in a cohort of 3980 men but only when considering the used PSA assay [17]. Beside the nomogram developed by Karakiewicz et al. [8] (named as nomogram I), another nomogram model has been published by Kawakami et al. [18] (named nomogram II). The increasing use of these free available multivariate models for PCa detection in the internet (http://www.nomograms.org/ or http://www.charite.de/pcaberlin/ann5/ann5.html) is an important point [8, 19]. Other models using clinically relevant data also add substantial information for detecting PCa while avoiding unnecessary biopsies in patients with benign prostates [10–12, 20–22].

## 2. Materials and Methods

### 2.1. Patient Population

From May 2005 to June 2008 a total of 282 patients (101 with PCa and 181 with no evidence of malignancy (NEM)) were included in the trial (median age 66 years) because of either a suspicious DRE a PSA value between 4 and 10 ng/mL. All patients were referred by urologists for PCa screening. None of the included patients had a TRUS-guided biopsy nor a transurethral resection of the prostate before.

### 2.2. Clinical and Pathologic Evaluation

The Beckman Access PSA assay was used for 195 patients and the Roche Elecsys 2010 for 87 patients and clinical stage was defined according to the sixth edition of the American Joint Committee on Cancer Staging Manual [23]. Blood samples were taken before prostate manipulation and centrifuged within 2-3 h after venipuncture. Serum was analyzed on the same day. Twelve core systematic TRUS-guided biopsies were performed in all subjects as described elsewhere [24]. All biopsy specimens were histologically graded according to the Gleason grading system by two pathologists. Total prostate volume was calculated with the prolate ellipsoid formula (volume = 0.52 × length × width × height). A DRE finding nonsuspicious for cancer was defined as negative; and a finding suspicious for cancer as positive.

### 2.3. Data Analysis

Data from all 282 patients were applied to the online available ANN “ProstataClass” (named ANN-Charité) using both the Beckman Access and the Roche Elecsys tPSA and fPSA assays [19]. This ANN was built on 798 samples (468 PCa and 330 NEM) investigated retrospectively from archival sera collected between 2001 and 2004 [19].

The ANN model was constructed with the MATLAB Neural Network Toolbox (The Mathworks, Natick, Mass, USA). Feed-forward back-propagation networks were built in which the input layer consisted of five neurons for the variables tPSA, %fPSA, age, prostate volume, and DRE, with three neurons as hidden layer and one output neuron, ranged from 0 (low PCa risk) to 1 (high PCa risk). To get the best generalization of the ANN, we used Bayesian regularization. To avoid overfitting the number of epochs to train the network over the entire set of input patterns was limited to 5. To compare possible population effects on model differences, two other LR-based models [7, 17] built on external cohorts were applied to our cohort. The calibration of the nomograms as help to compare the predicted and observed probabilities was performed as described before [19]. However, the282 patients were subdivided in 10 groupsof each28 men in order of their respective predicted nomogram probability. For each group the observed and mean predicted probabilities were computed.

### 2.4. Selection of Two Other Nomograms

Nomogram I was developed by Karakiewicz et al. [8] nomogram II and was published by Kawakami et al. [18]. Both nomograms had very similar patients' characteristics regarding the number of included patients. Karakiewicz's nomograms belong to the mostly used nomograms in the internet. These are reasonable facts for us to select these nomograms to compare the results with our population.

### 2.5. Statistical Analyses

All 282 observations were used to access the predictive accuracy and the performance characteristics of the ANN [19]. The individual ANN predictions were generated with the use of the web-based ANN application, which relies on age, DRE, PSA, %fPSA, and prostate volume.

We used the statistical software SPSS 17.0 for Windows (SPSS, Chicago, USA) and Sigma Plot 2001 for Windows. The nonparametric Kruskal-Wallis test of variance, the Mann-Whitney *U* test, logistic regression analysis with forward variable section, and Spearman rank correlation were carried out. The diagnostic validity of tPSA, %fPSA, and the ANN was evaluated by ROC curve analysis with calculations of the AUC and specificities at 90% and 95% sensitivity by using Graph ROC for Windows [25] and MedCalc 11.2.1 (MedCalc Software, Mariakerke, Belgium). Significance was defined at *P* < 0.05.

## 3. Results

Table 1 shows the characteristics of the cohort of 282 patients used in the external validation of the ANN. Age ranged between 46 and 83 years (median: 66). In the Beckman Group PSA and %fPSA ranged from 4.01 to 9.91 ng/mL (median: 6.77) and 5% to 48% (median: 15.69%), respectively. In the Roche Group PSA and %fPSA ranged from 4.01 to 9.99 ng/mL (median: 6.98) and 4% to 31% (median: 15.63%), respectively. Of all men, 67 (23.8%) demonstrated suspicious DRE findings. Total prostate volume ranged from 7.1 to 171.0 cc (median: 42.6). Overall, 101 (35.8%) PCa were detected. Of men with suspicious DRE, 37 (55.2%) had PCa on biopsy.

Table 2 shows median and mean values for age, tPSA, %fPSA, prostate volume, and DRE status for the validation cohort and the training cohort for the ANN-Charité. The percentage of PCa patients (35.8%) is much lower in our external validation cohort as in the “ProstataClass” cohort (58.6%). Comparisons between PCa and NEM within our external validation cohort and the “ProstataClass” cohort revealed significant differences for age, %fPSA, PSAD, and number of positive DREs (*P* always <0.05) with exception for tPSAs (*P* = 0.387) and volume (*P* = 0.900) in the external validation cohort.

**Figure 1:**
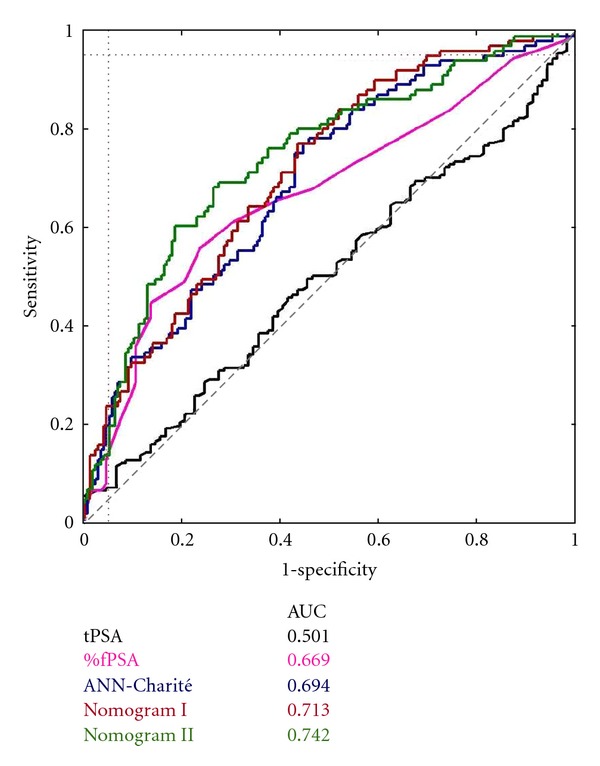
ROC curves.

The ANN, which is based on age, DRE, PSA, %fPSA, and prostate volume, was 78% accurate in the original report [10]. As shown in Figure 1, ROC curve analyses for tPSA, %fPSA, and the ANN were performed for our cohort. The AUCs of ROC curve analysis were 0.501 for tPSA, 0.669 for %fPSA, 0.694 for ANN-Charité, 0.713 for nomogram I, and 0.742 for nomogram II, showing a significant advantage for the nomogram II (*P* = 0.009) compared with %fPSA while the other models did not differ from %fPSA (*P* = 0.15 and *P* = 0.41). The ROC analyses also demonstrated a higher specificity at 95% sensitivity for nomogram I (specificity 30.4%) compared with %fPSA (specificity 12.9%), tPSA (specificity 3.96%), nomogram II (specificity 18.2%), or ANN-Charité (specificity 18.8%). At 90% sensitivity the ROC analyses demonstrated a higher specificity for nomogram I (specificity 40.9%) compared with %fPSA (specificity 25.7%), tPSA (specificity 6.93%), nomogram II (specificity 27.1%), or ANN-Charité (specificity 33.7%). These data at 90% and 95% sensitivity confirm the similarities between ANN models and nomograms.

**Figure 2:**
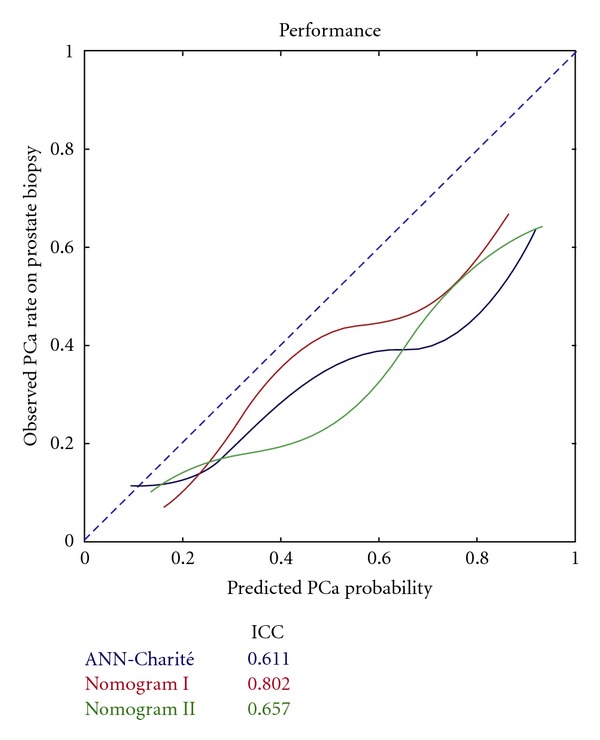
Performance of predicted PCa probability.

Beside ROC analysis, the concordance between the predicted PCa and observed PCa probability is a good measure of a multivariate model's quality. In Figure 2, the predicted PCa probabilities are shown in relation to the observed PCa rate for the ANN model and nomograms. In the case of total concordance, there is no difference between predicted and observed probabilities—all points lie on the 45° line. Here the intraclass correlation coefficient (ICC) is a measure for the consistence of the observed and predicted values and a value of 1 would be ideal. To suppress random fluctuations in graphical representation a cubic smoothing spline was computed to expose the relationship between predicted and observed probabilities. The intraclass correlation coefficients for the observed versus predicted probabilities were 0.802 for nomogram I, 0.611 for the ANN-Charité, and 0.657 for nomogram II. 

We further performed the decision curve analysis and found only marginal differences between the 3 models.

## 4. Discussion

In the “ProstataClass” cohort the indications for referral were increased PSA values, lower urinary tract symptoms, abnormal DRE, or biopsy confirmed PCa, which explains the higher number of PCa patients [10, 19]. Our population is a screening population for PCa, and only suspicious DRE and/or a PSA value between 4.01 and 9.99 ng/mL were indications for biopsy. This could be a reason why our detection rate is lower than in the original cohort from Charité Universitätsmedizin Berlin [19]. The ANN-Charité was created for a PSA range 0–27 ng/mL; so it can also be applied for the PSA range 4–10 ng/mL we used in our cohort.

Different molecular forms of PSA, PSA density and velocity, or age-adjusted cutoffs ameliorate the detection rates in screening for PCa [4]. It has been shown that the use of %fPSA significantly improves specificity by ~15–20% compared with tPSA [10, 11, 26]. The AUC for %fPSA in our cohort (0.669) runs significantly above the AUC for tPSA (0.501). Our data confirm the improved diagnostic accuracy of %fPSA. The AUCs for ANN-Charité (0.694), nomogram I (0.713), and nomogram II (0.742) were all above the %fPSA AUC, but only nomogram II reached significance. When evaluating the specificities at the clinical important cutoff of 95% sensitivity, surprisingly the nomogram I was superior compared with %fPSA, tPSA, nomogram II, and ANN-Charité. However, these results show the clinical importance of cutoffs when using the ANN model or a nomogram instead of a single %fPSA or tPSA cutoff for biopsy decision. It should be mentioned that published ANN models mostly provide cutoffs for a biopsy decision [11, 19] while published nomograms usually estimate a PCa probability only [8, 16, 18]. For external user a given cutoff for biopsy decision is easier to handle and should be therefore preferred.

Data on PSA-assay-specific comparisons of different ANN models and nomograms regarding retrospective and prospective data generation are rare. As seen in Tables 2 and 3, one of the main aims of this study could be reached only partially since the ANN-Charité could not repeat its significantly better performance compared with %fPSA in our cohort. Possible reasons for this relatively weak performance of the ANN-Charité are already provided when comparing our cohort and the “Prostata Class” cohort.

In 2007, Stephan et al. were able to show that different ANN and LR models perform similarly when applying to the same cohort [27]. This hypothesis was clearly confirmed in this study where ANN-Charité and the nomograms performed similarly, but not the same, when testing them in the same cohort.

We have differences between the ANN-Charité (AUC = 0.694) and the nomograms. This could be caused by differences in PCa detection rate, age, %fPSA, PSA, and number of positive DREs in the training and test cohort. While the overall ANN performance in the “ProstataClass” cohort was superior compared with the other cohort, the AUC difference between tPSA and the ANN models is smaller (<0.2) in the “ProstataClass” cohort compared with our cohort (0.18 to 0.25). This is mainly due to the large AUC of tPSA in the “ProstataClass” cohort with already 0.7. However, several other points showed a good comparability between the original and our cohort. The percentage of tPSA and prostate volume did not differ between both cohorts. The prostate volume is an important variable for this ANN. In our study, we used two systematic sextant patterns to take the biopsies in all cases. In other studies, it was shown that in patients with larger prostate volume a higher number of biopsies is useful. This should be considered as a yield of the prostate cancer detection rate [26]. Especially in the cohort of Kawakami et al., the number of biopsy cores was much higher with 20 cores in mean [18]. Furthermore, the typical significance between PCa and NEM patients was visible in both cohorts.

When analyzing Figures 1 and 2, the AUC differences appear small, but the calibration curves and ICC differences are larger. The results from analyzing the Saarow cohort with the ANN-Charité failed to show an improved performance with an AUC of 0.694 and an ICC of 0.611 only. While the two nomograms showed smaller differences in their AUC values, the differences in their performance were large when comparing the calibration curves and ICC. Thus, when only analyzing AUC values in validation studies, differences in predicted and observed PCa detection rates may not be detected [20].

Stephan et al. [10] could show in the first multicenter evaluation in almost 1200 men within a broad PSA range of 2–20 ng/mL that the combination of age, DRE, PSA, %fPSA, and prostate volume clearly enhances the specificity of %fPSA by 20% at 95% sensitivity. However, this ANN was built only with one PSA and fPSA assay (Immulite 2000 systems, Siemens Healthcare Diagnostics). By using a new model of this ANN built on 5 different tPSA and fPSA assays [19] we could show that this ANN by using the Beckman Coulter Access PSA assay confirms the diagnostic improvements. Using multivariable models has several advantages over using a single parameter for important clinical decisions and is seen as one of the future ways to maximize specificity for PCa detection [15]. We believe that paper versions of models like nomograms could not be as practical as internet- or computer-based nomogram models or ANN programs like “ProstataClass” [19] or the ANN by Finne et al. [11]. Web- or computer based software is needed to integrate such models in clinical practice.

Regardless of the method used, nomograms and especially ANN help to assess the patient's risk of PCa better than single parameters like %fPSA, complexed PSA, or PSA alone. Using this recently introduced ANN [19] the number of unnecessary biopsies can be reduced.

## 5. Conclusion

Our results showed limitations of multivariate models when external validations were performed without keeping in mind the circumstances of the model development especially in population characteristics. However, models like the used ANN are more helpful in daily routine to increase the PCa detection rate and reduce unnecessary biopsies compared with nomograms used due to the usability of cutoffs.

## Figures and Tables

**Table 1:** Patients' characteristics.

Characteristic	Beckman Group	Roche Group
Patients [*n*]	195	87
Age [years]		
Mean	66.10	67.39
Median	66	67
Range	46–83	50–81
PSA [ng/mL]		
Mean	6.78	6.98
Median	6.65	6.89
Range	4.01–9.91	4.01–9.99
%fPSA		
Mean	15.69	15.63
Median	15	14
Range	5–48	4–31
Suspicious DRE [*n*]	48 (24.6%)	19 (21.8%)
Total prostate volume [cc]		
Mean	40.09	48.29
Median	36.0	43.8
Range	7.1–119.2	11.5–171.0
Prostate cancer on needle biopsy [*n*]	71 (36.4%)	30 (34.5%)

**Table 2:** Mean and median values with ranges for age, tPSA, %fPSA, prostate volume, PSAD, and number of positive (suspicious) DREs in all, PCa, and NEM patients for both cohorts.

Variable	External validation cohort			“ProstataClass” cohort^+^			*P* value
	All *n* = 282	PCa *n* = 101	NEM *n* = 181	All *n* = 798	PCa *n* = 468	NEM *n* = 330
Age (years)							
Mean	66	67	67	64	63	66	
Median	67^∗^	68^∗^	66^∗^	64	63	66	<0.0005
Range	46–83	50–82	46–83	38–85	43–79	38–85	
tPSA (ng/mL)							
Mean	6.84	6.83	6.85	7.54	8.77	5.81	
Median	6.66	6.66	6.79	6.63	7.75	4.69	0.387
Range	4.01–9.99	4.01–9.61	4.06–9.99	0.49–27.04	0.86–24.02	0.49–27.04	
%fPSA (%)							
Mean	15.67	13.14	17.1	14.84	11.30	19.86	
Median	15.0^∗^	12.0^∗^	16.0^∗^	12.75	10.27	18.31	0.002
Range	4.0–48.0	5.0–30.0	4.0–48.0	2.52–69.39	3.10–49.86	2.52–69.39	
Volume (mL)							
Mean	42.62	34.14	47.35	43.7	37.2	52.9	
Median	37.65	29.9	43.8	38	34	45.5	0.900
Range	7.1–171	7.1–100	15–171	10–180	10–110	13–180	
PSAD (tPSA/Vol.)							
Mean	0.197	0.242	0.172	0.211	0.276	0.120	
Median	0.174	0.221	0.154	0.157	0.224	0.086	0.030
Range	0.04–0.80	0.05–0.80	0.04–0.55	0.01–1.35	0.03–1.35	0.01–1.00	
No. pos. DRE (%)	67 (23.8%)^∗^	37 (36.6%)^∗^	30 (16.6%)^∗^	314 (39.3%)	284 (60.7%)	30 (9.1%)	<0.0005

^+^PSA and %fPSA of “ProstataClass” cohort using the Beckman Access assay.

^∗^Significantly different from “ProstataClass” cohort.

**Table 3:** ROC curve analysis for tPSA, %fPSA, ANNs, and nomograms.

Parameter cohort	tPSA	%fPSA	Karakiewicz et al.'s Nomogram I	Kawakami et al.'s Nomogram II	ANN-Charité
Area under the ROC curve (AUC)					

Saarow cohort	0.501	0.669	0.713	0.742^∗^	0.694
“ProstataClass” cohort	0.7	0.782			

Specificity at 95% sensitivity					

Saarow cohort	3.96%	12.9%	30.4%	18.2%	18.8%
“ProstataClass” cohort	27.8%	27.5%			

Specificity at 90% sensitivity					

Saarow cohort	6.93%	25.7%	40.9%	27.1%	33.7%
“ProstataClass” cohort	39.4%	44.1%			

^∗^Significantly different from %fPSA.
